# Efficacy of Filgotinib in Patients with Ulcerative Colitis by Line of Therapy in the Phase 2b/3 SELECTION Trial

**DOI:** 10.1093/ecco-jcc/jjad039

**Published:** 2023-03-16

**Authors:** Iris Dotan, Brian G Feagan, Virginia Taliadouros, Alessandra Oortwijn, Christine Rudolph, Angela de Haas, Eva Santermans, Jeremy Hsieh, Laurent Peyrin-Biroulet, Toshifumi Hibi

**Affiliations:** Division of Gastroenterology, Rabin Medical Center, Petah Tikva, Israel; Sackler Faculty of Medicine, Tel Aviv University, Tel Aviv, Israel; Alimentiv, London, ON, Canada; Division of Gastroenterology, London Health Sciences Centre, Western University, London, ON, Canada; Galapagos NV, Leiden, The Netherlands; Galapagos NV, Leiden, The Netherlands; Galapagos NV, Leiden, The Netherlands; Galapagos NV, Mechelen, Belgium; Galapagos NV, Mechelen, Belgium; Gilead Sciences, Inc., Foster City, CA, USA; University of Lorraine, Inserm, NGERE, Nancy, France; Groupe Hospitalier Privé Ambroise Paré – Hartmann, Paris IBD Center, Neuilly sur Seine, France; Center for Advanced IBD Research and Treatment, Kitasato University Kitasato Institute Hospital, Tokyo, Japan

**Keywords:** Filgotinib, ulcerative colitis, positioning

## Abstract

**Background and Aims:**

The efficacy of new therapies for ulcerative colitis [UC] is usually influenced by previous biologic use. These *post hoc* analyses of SELECTION, a placebo-controlled phase 2b/3 trial in patients with moderately to severely active UC, evaluated the efficacy of filgotinib, an oral Janus 1 kinase preferential inhibitor, with respect to prior biologic failure.

**Methods:**

The effect of filgotinib 200 mg (FIL200) relative to placebo was compared in biologic-naïve and biologic-failed patient groups, and in further subgroups by number of failed biologics [1 or >1], biologic mechanism of action [MoA] classes [1 or 2] and tumour necrosis factor [TNF] antagonists [1 or >1]. Odds ratios [ORs] for clinical remission at week 10 [induction] and hazard ratios [HRs] for protocol-specific disease worsening [PSDW] from week 11 to week 58 [maintenance] were calculated.

**Results:**

At week 10, FIL200-treated patients were more likely to achieve clinical remission than placebo-treated patients in the biologic-naïve (OR [95% confidence interval, CI]: 1.98 [1.14–3.44]) and biologic-failed (3.91 [1.33–11.48]) groups. During maintenance, FIL200-treated patients had a reduced risk of PSDW in the biologic-naïve (HR [95% CI]: 0.22 [0.11–0.44]) and biologic-failed (0.22 [0.12–0.40]) groups, and in all biologic-failed subgroups (except >1 TNF antagonist failure). The data suggest that the likelihood of PSDW at week 58 increased with increasing numbers of failed biologics.

**Conclusions:**

FIL200 induced and maintained benefits relative to placebo regardless of previous biologic use; however, the estimated therapeutic benefit was greatest in biologic-naïve patients and patients previously treated with one biologic or biologic MoA class. [ClinicalTrials.gov: NCT02914522].

## 1. Introduction

Ulcerative colitis [UC] is an immune-mediated chronic disease characterized by mucosal inflammation, manifested by bloody diarrhoea and frequent bowel movements.^1^ The short- and intermediate-term treatment goals for patients with UC include achieving clinical remission and a reduction in inflammation as assessed by biomarkers and endoscopy.^2^ Longer-term goals of therapy include corticosteroid-free remission, mucosal healing, normalized quality of life, and prevention of surgery and colorectal cancer.^2^ Available treatments for moderately to severely active UC include: corticosteroids; immunosuppressants such as thiopurines and calcineurin inhibitors; tumour necrosis factor [TNF] antagonists; the anti-integrin vedolizumab; the interleukin-12/23 inhibitor ustekinumab; the sphingosine-1-phosphate modulator ozanimod; and the Janus kinase [JAK] inhibitors tofacitinib, upadacitinib and filgotinib. The recently introduced JAK inhibitors and ozanimod are targeted small-molecule oral drugs.^3,4^ Of the JAK inhibitors, tofacitinib is a pan-JAK inhibitor, inhibiting JAK1, JAK2 and JAK3, upadacitinib is a JAK1 and JAK1/3 preferential inhibitor, whereas filgotinib is a preferential JAK1 inhibitor.^5–8^

The increasing number of approved treatment options for UC, and the increasing availability of biosimilars and even generics, means that most new therapies will be used as second- or third-line therapies. A network meta-analysis of UC clinical trials suggests that clinical remission at induction occurs more frequently in patients using biologics as first-line therapy than in patients with prior exposure to TNF antagonists.^9^ However, the efficacy of first-line biologics is limited, and there are limited evidence-based recommendations for managing primary non-response to TNF antagonists by switching to a different drug class.^10,11^ Furthermore, the UC patient populations that are included in clinical trials have become increasingly refractory to treatment.^12^ In parallel, clinical trial design has evolved to reflect these changes, in order to demonstrate efficacy in these more refractory patient populations.^12^ In this context, it is important to understand the impact of previous biologic use on the treatment efficacy of the recently approved small molecules, including JAK inhibitors.

SELECTION was a phase 2b/3 trial of filgotinib in biologic-naïve and biologic-experienced patients with moderately to severely active UC.^13^ Filgotinib 200 mg was shown to be well tolerated and effective in inducing and maintaining clinical remission compared with placebo.^13^ In these *post hoc* analyses of SELECTION data, we aimed to explore the efficacy of filgotinib in biologic-naïve patients compared with those in whom biologic therapy had previously failed. We also assessed the efficacy of filgotinib in the subgroups of patients with failure of one or more than one biologic, one or two biologic mode of action [MoA] classes [TNF antagonist and vedolizumab], and one or more than one TNF antagonist.

## 2. Methods

### 2.1. Study design

SELECTION [ClinicalTrials.gov ID: NCT02914522] was a phase 2b/3, double-blind, randomized, placebo-controlled trial comprising two induction studies and a maintenance study. Details of the study design have been previously described by Feagan *et al*.^13^ Eligible patients with moderately to severely active UC were enrolled into one of two induction studies: Induction Study A [biologic-naïve patients] and Induction Study B [biologic-experienced patients]. Moderately to severely active UC was defined as a Mayo endoscopic subscore [MES] of ≥2, rectal bleeding [RB] subscore of ≥1, stool frequency [SF] subscore of ≥1 and physician’s global assessment [PGA] subscore of ≥2; these subscores sum to a total Mayo Clinic Score [MCS] of 6–12. In each of the induction studies, patients were randomized 2:2:1 to receive filgotinib 200 mg, filgotinib 100 mg or placebo orally once daily for 11 weeks. Patients who received filgotinib and achieved either clinical remission or an MCS response at week 10 were re-randomized 2:1 at week 11 to continue their induction filgotinib regimen or to receive placebo in the maintenance study through to week 58. Placebo induction responders continued receiving placebo in the maintenance study. Clinical remission was defined as an MES of 0 or 1, RB subscore of 0, and a ≥1 point decrease in SF subscore from induction baseline to achieve a subscore of 0 or 1. An MCS response was defined as a reduction of ≥3 points in MCS that resulted in an MCS score that was ≥30% smaller compared with induction baseline, with an accompanying decrease in RB subscore of ≥1 point, or an absolute RB subscore of 0 or 1.

SELECTIONLTE [ClinicalTrials.gov ID: NCT02914535] is an extension study evaluating the long-term safety of filgotinib.^14^ The study enrolled patients who completed or met protocol-specified efficacy discontinuation criteria in SELECTION. Efficacy discontinuation criteria in SELECTION comprised either non-response to treatment [assessed at week 10 of the induction study] or disease worsening during the maintenance study. Patients who discontinued SELECTION were offered open-label filgotinib 200 mg in SELECTIONLTE. The exception to this was men in the USA for whom two prior biologic MoA classes had failed [TNF antagonist and vedolizumab], who received filgotinib 100 mg instead.

Both studies were carried out in accordance with the International Conference on Harmonisation Good Clinical Practice Guidelines and the Declaration of Helsinki. All patients provided written informed consent before study inclusion.

### 2.2. Outcome measures and assessments

In these *post hoc* analyses of SELECTION, biologic-naïve and biologic-failed patient groups were compared. The biologic-failed group was defined as a subset of biologic-experienced patients for whom treatment failure was given as a reason for discontinuation of biologic therapy. Accordingly, patients with intolerance as a reason for biologic discontinuation were not included in the biologic-failed group. The biologic-failed group was further divided into subgroups of those who had failure of: one or more than one prior biologic; one or two biologic MoA classes [TNF antagonist and vedolizumab]; and one or more than one TNF antagonist exclusively. The individual TNF antagonists considered for analysis were adalimumab, golimumab and infliximab. The number of patients who had failure of vedolizumab exclusively was too small to be a subgroup in which efficacy could feasibly be analysed in these *post hoc* analyses. We assessed the proportions of patients who achieved either clinical remission or an MCS response in the biologic-naïve and biologic-failed groups, as well as in the biologic-failed subgroups. Furthermore, for each induction treatment arm in the biologic-naïve and biologic-failed groups, the rapidity of response during the first 15 days of the induction studies was assessed using the criterion of an RB subscore of 0. We also measured the time to protocol-specified disease worsening [PSDW] during the maintenance study among patients in all groups and subgroups. PSDW, as previously described by Feagan *et al.*, was assessed starting at week 11 based on the partial MCS [pMCS], which includes all components of the MCS except for the endoscopic subscore.^13^ PSDW was defined as an increase in pMCS of ≥3 points to at least 5 points from the week 10 value on two consecutive visits, or an increase to 9 points on two consecutive visits if the week 10 value was >6.^13^ Finally, we measured the change in pMCS from induction baseline through to week 58 in the biologic-naïve and biologic-failed groups, considering a patient population that combined patients treated with filgotinib 200 mg throughout SELECTION and filgotinib 200 mg non-responders who entered SELECTIONLTE.

### 2.3. Statistical analysis

The analyses were conducted using the full analysis set for data derived from the induction and maintenance phases of SELECTION, and using the safety analysis set for data from patients who entered the SELECTIONLTE study.

In the induction studies, the rapidity of response [RB subscore of 0] was assessed using Kaplan–Meier estimates. Relative differences in efficacy [clinical remission and MCS response] at week 10 between filgotinib 200 mg, filgotinib 100 mg and placebo in the biologic-naïve and biologic-failed groups, and in the biologic-failed subgroups, were estimated using odds ratios [ORs] and 95% confidence intervals [CIs]. ORs and 95% CIs were derived using a logistic regression model with adjustment for the following variables at induction baseline: concomitant use of oral, systemic corticosteroids; concomitant use of immunosuppressives; C-reactive protein; and sex.

In the maintenance study, PSDW was assessed using Kaplan–Meier estimates. A Cox proportional hazards model was used to estimate the hazard ratios [HRs] and 95% CIs for filgotinib 200 mg and filgotinib 100 mg relative to placebo. The model was stratified according to concomitant use of oral, systemic corticosteroids and of immunosuppressives, an RB subscore of 0, and faecal calprotectin concentration at maintenance baseline. HRs were calculated for all groups and subgroups. The relative differences in efficacy at week 58 between filgotinib 200 mg, filgotinib 100 mg and placebo were estimated using ORs and 95% CIs derived using a logistic regression model with adjustment for the stratification variables. Additionally, least-squares mean [95% CI] changes in pMCS from induction baseline to week 58 were determined using a mixed model for repeated measures [MMRM] for biologic-naïve and biologic-failed patients who received filgotinib 200 mg. The MMRM included concomitant use of oral, systemic corticosteroids and of immunosuppressives, MES and pMCS, and disease duration at induction baseline.

For the assessment of clinical remission and MCS response at weeks 10 and 58, non-responder imputation was used. For the assessment of rapidity of response, patients were censored at the last available RB subscore in the 15-day window. For the assessment of time to PSDW, patients were censored at the last non-missing PGA assessment for the pMCS, before treatment failure, if it occurred. Patients who discontinued treatment but continued participation in the study (and therefore continued to have PGA assessments) were not censored. No imputation was used for the assessment of change in pMCS from induction baseline, given the MMRM handled missing data under the missing-at-random assumption. All statistical analyses were performed in SAS 9.4 (SAS Institute). As these were *post hoc* analyses, and no adjustment was made for multiplicity, all reported statistical significances in terms of clinical remission, MCS response and PSDW are nominal.

## 3. Results

### 3.1. Patient disposition and baseline characteristics

The induction studies included a total of 665 biologic-naïve patients and 683 biologic-experienced patients, of whom 649 were patients with biologic failure. Overall, 323 filgotinib-treated biologic-naïve patients and 218 filgotinib-treated patients with biologic failure entered the maintenance study [Supplementary Figure 1]. Induction baseline and disease characteristics for the biologic-naïve and biologic-failed patient groups are shown in Table 1. Among the biologic-failed patient group, the induction baseline and disease characteristics for the subgroups by number of failed biologics, biologic MoA classes and TNF antagonists are shown in Supplementary Table 1. Patient baseline characteristics were generally similar across treatment groups and subgroups. Nonetheless, a numerically greater proportion of patients in the biologic-naïve group than in the biologic-failed group were Asian across the induction treatment arms (31.0, 28.2 and 27.9% vs 18.7, 16.6 and 19.7%; filgotinib 200 mg, filgotinib 100 mg and placebo, respectively). Additionally, there was a numerically higher proportion of patients in the biologic-failed group than in the biologic-naïve group with a history of smoking (28.0, 32.8 and 31.1% vs 22.2, 19.5 and 16.4%; filgotinib 200 mg, filgotinib 100 mg and placebo, respectively). For patients with biologic failure, disease characteristics generally indicated greater disease burden for patients in whom multiple biologics had failed compared with patients in whom a single biologic agent had failed. For example, the proportions of patients with an MES of 3 were 71.4, 74.1 and 74.5% in the subgroup with failure of one biologic and 83.1, 81.8 and 81.5% in the subgroup with failure of more than one biologic, for filgotinib 200 mg, filgotinib 100 mg and placebo, respectively. Among the biologic-failed group, infliximab was the TNF antagonist that had failed in the greatest proportion of patients (62.2, 64.9 and 60.6%; filgotinib 200 mg, filgotinib 100 mg and placebo, respectively), whereas golimumab and adalimumab had failed in ~20 and 50% of patients, respectively. More than half of patients in the biologic-failed group had previous failure of vedolizumab (58.5, 47.2 and 56.1%; filgotinib 200 mg, filgotinib 100 mg and placebo, respectively). Similarly, approximately half of patients in the biologic-failed group had previous failure of both vedolizumab and TNF antagonists (48.8, 42.1 and 48.5%). Therefore, the sample size for patients who only had vedolizumab failure was small.

**Table 1.** Patient induction baseline and disease characteristics for the biologic-naïve and biologic-failed groups.

	Biologic-naïve			Biologic-failed		
	Filgotinib 200 mg [*n* = 248]	Filgotinib 100 mg [*n* = 277]	Placebo [*n* = 140]	Filgotinib 200 mg [*n* = 246]	Filgotinib 100 mg [*n* = 271]	Placebo [*n* = 132]
Age, years, mean ± SD	42.4 ± 13.0	42.3 ± 13.3	41.5 ± 12.8	43.4 ± 14.1	42.8 ± 14.2	44.2 ± 15.1
Sex, female, *n* [%]	123 [49.6]	121 [43.7]	51 [36.4]	102 [41.5]	91 [33.6]	52 [39.4]
Race, *n* [%]						
American Indian or Alaska native	1 [0.4]	0	0	0	0	0
Asian	77 [31.0]	78 [28.2]	39 [27.9]	46 [18.7]	45 [16.6]	26 [19.7]
Black or African-American	2 [0.8]	3 [1.1]	1 [0.7]	4 [1.6]	6 [2.2]	3 [2.3]
White	168 [67.7]	193 [69.7]	96 [68.6]	178 [72.4]	204 [75.3]	90 [68.2]
Other	0	2 [0.7]	2 [1.4]	0	0	1 [0.8]
Not permitted^a^	0	1 [0.4]	2 [1.4]	18 [7.3]	16 [5.9]	12 [9.1]
Ethnicity, *n* [%]						
Not Hispanic or Latino	241 [97.2]	269 [97.1]	137 [97.9]	234 [95.1]	259 [95.6]	124 [93.9]
Hispanic or Latino	6 [2.4]	6 [2.2]	3 [2.1]	7 [2.8]	8 [3.0]	4 [3.0]
Not permitted^a^	1 [0.4]	2 [0.7]	0	5 [2.0]	4 [1.5]	4 [3.0]
Smoking status, *n* [%]						
Former	55 [22.2]	54 [19.5]	23 [16.4]	69 [28.0]	89 [32.8]	41 [31.1]
Current	15 [6.0]	11 [4.0]	5 [3.6]	7 [2.8]	20 [7.4]	5 [3.8]
Never	178 [71.8]	212 [76.5]	112 [80.0]	170 [69.1]	162 [59.8]	86 [65.2]
Duration of UC from diagnosis, years, mean ± SD	7.2 ± 7.0	6.7 ± 7.4	6.5 ± 7.4	9.8 ± 7.5	9.6 ± 7.1	10.1 ± 8.3
MCS, mean ± SD	8.6 ± 1.3	8.6 ± 1.4	8.7 ± 1.3	9.3 ± 1.4	9.3 ± 1.3	9.3 ± 1.4
Mayo endoscopic subscore of 3, *n* [%]	135 [54.4]	159 [57.4]	78 [55.7]	193 [78.5]	213 [78.6]	104 [78.8]
Faecal calprotectin, μg/g, mean ± SD	2044 ± 2627	1897 ± 3046	2208 ± 2891	2818 ± 4006	2382 ± 3538	2531 ± 3671
Concomitant use of corticosteroids on day 1, *n* [%]	75 [30.2]	88 [31.8]	42 [30.0]	117 [47.6]	123 [45.4]	60 [45.5]
Prior use of adalimumab, *n* [%]	0	0	0	130 [52.8]	142 [52.4]	72 [54.5]
Prior use of golimumab, *n* [%]	0	0	0	56 [22.8]	54 [19.9]	27 [20.5]
Prior use of infliximab, *n* [%]	0	0	0	184 [74.8]	202 [74.5]	96 [72.7]
Prior use of vedolizumab, *n* [%]	0	0	0	162 [65.9]	142 [52.4]	83 [62.9]
Prior failure of adalimumab, *n* [%]	0	0	0	119 [48.4]	133 [49.1]	69 [52.3]
Prior failure of golimumab, *n* [%]	0	0	0	49 [19.9]	50 [18.5]	26 [19.7]
Prior failure of infliximab, *n* [%]	0	0	0	153 [62.2]	176 [64.9]	80 [60.6]
Prior failure of vedolizumab, *n* [%]	0	0	0	144 [58.5]	128 [47.2]	74 [56.1]
Prior use of both TNF antagonist^b^ and vedolizumab, *n* [%]	0	0	0	146 [59.3]	127 [46.9]	75 [56.8]
Prior failure of both TNF antagonist and vedolizumab, *n* [%]	0	0	0	120 [48.8]	114 [42.1]	64 [48.5]

^a^Local regulators did not allow collection of race and/or ethnicity information.

^b^TNF antagonists included adalimumab, golimumab and infliximab.

MCS, Mayo Clinic Score; SD, standard deviation; TNF, tumour necrosis factor; UC, ulcerative colitis.

### 3.2. Rapidity of response, clinical remission and MCS response at week 10

**Figure 1.**
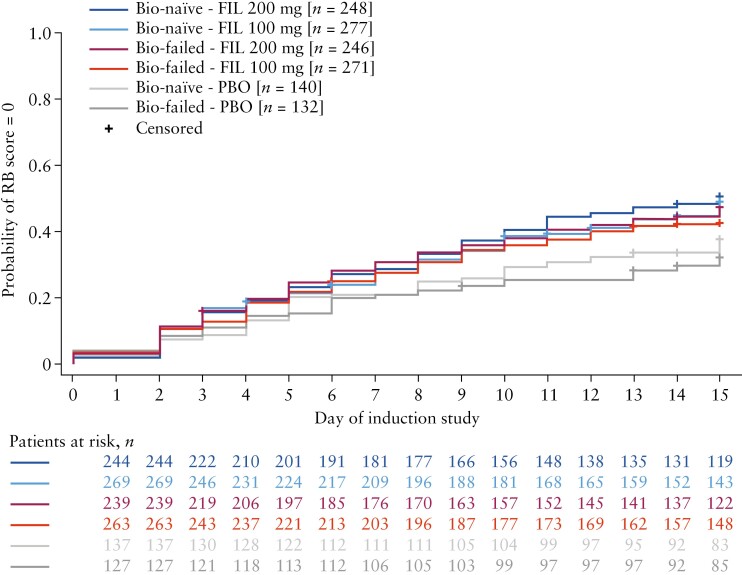
Rapidity of response in terms of RB subscore of 0 during the first 15 days of induction studies in the biologic-naïve and biologic-failed groups by treatment arm. Bio, biologic; FIL, filgotinib; PBO, placebo; RB, rectal bleeding.

**Figure 2.**
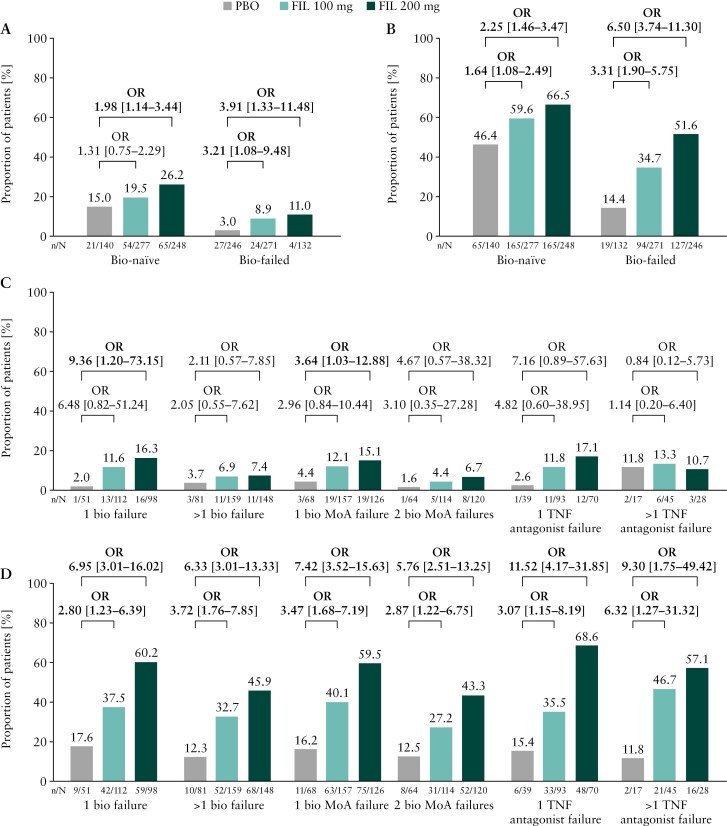
Proportions of biologic-naïve and biologic-failed patients [A] in clinical remission and [B] with an MCS response at week 10, and proportions of patients by number of failed biologics, biologic MoA classes and TNF antagonists [C] in clinical remission and [D] with an MCS response at week 10. ORs [95% CI] are given in terms of FIL 100 mg or FIL 200 mg vs placebo in each of the groups and subgroups. ORs [95% CI] given in bold type indicate statistical significance. Bio, biologic; CI, confidence interval; FIL, filgotinib; MCS, Mayo Clinic Score; MoA, mechanism of action; OR, odds ratio; PBO, placebo; TNF, tumour necrosis factor.

In patients receiving filgotinib 200 mg, similar kinetics were observed during the first 15 days of induction therapy with respect to achieving an RB subscore of 0 in biologic-naïve and biologic-failed patients [Figure 1]. Consistent with this observation, both biologic-naïve and biologic-failed patients treated with filgotinib 200 mg were nominally significantly more likely to achieve clinical remission (OR [95% CI]: biologic-naïve, 1.98 [1.14–3.44]; biologic-failed, 3.91 [1.33–11.48]) [Figure 2A] and an MCS response (OR [95% CI]: biologic-naïve, 2.25 [1.46–3.47]; biologic-failed, 6.50 [3.74–11.30]) [Figure 2B] at week 10 compared with placebo. Among biologic-failed patients receiving filgotinib 200 mg, patients in the subgroups of one biologic failure and one biologic MoA class failure were nominally significantly more likely to achieve clinical remission relative to placebo, with ORs [95% CIs] of 9.36 [1.20–73.15] and 3.64 [1.03–12.88], respectively [Figure 2C]. Filgotinib 200 mg-treated patients with failure of more than one biologic, two biologic MoA classes or one TNF antagonist were also more likely to achieve clinical remission compared with placebo, though these results were not nominally statistically significant. Filgotinib 200 mg-treated patients with failure of more than one TNF antagonist were not more likely to achieve clinical remission [Figure 2C]. Patients receiving filgotinib 200 mg were nominally significantly more likely to achieve an MCS response compared with placebo, regardless of the number of previously failed biologics or biologic MoA classes [Figure 2D].

### 3.3. Clinical remission and MCS response at week 58

Similar to the week 10 findings, at the end of maintenance, week 58, both biologic-naïve and biologic-failed patients treated with filgotinib 200 mg during maintenance were nominally significantly more likely to achieve clinical remission (OR [95% CI]: biologic-naïve, 6.18 [2.69–14.20]; biologic-failed, 6.04 [1.31–27.79]) [Supplementary Figure 2A] and an MCS response (OR [95% CI]: biologic-naïve, 4.90 [2.41–9.95]; biologic-failed, 5.86 [2.35–14.57]) [Supplementary Figure 2B] compared with patients who were re-randomized to placebo. Among biologic-failed patients who received filgotinib 200 mg during maintenance, patients in all but one subgroup [more than one TNF antagonist failure] were more likely to achieve clinical remission compared with patients who were re-randomized to placebo, although these results were not nominally statistically significant [Supplementary Figure 2C]. For MCS response, patients who received filgotinib 200 mg during maintenance in all biologic-failed subgroups apart from one [more than one TNF antagonist failure] were nominally significantly more likely to achieve an MCS response compared with patients who were re-randomized to placebo [Supplementary Figure 2D].

### 3.4. Time to PSDW during maintenance

**Figure 3.**
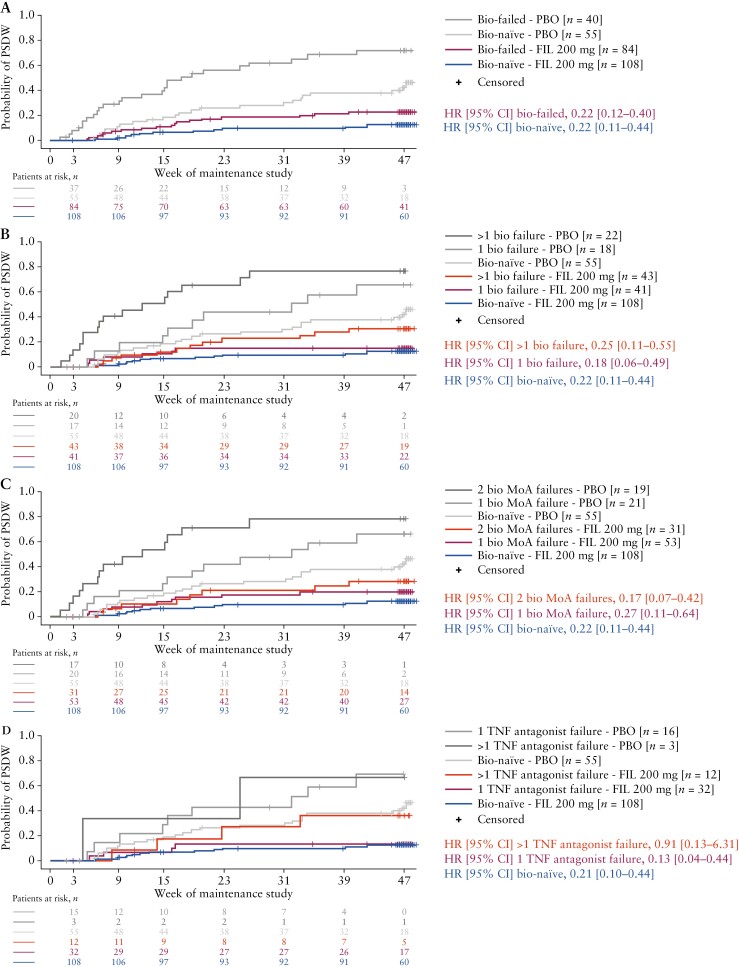
Time to PSDW for patients treated with filgotinib 200 mg during induction [A] in biologic-naïve and biologic-failed patients, and among biologic-failed patients categorized by number of [B] biologic failures, [C] biologic MoA class failures and [D] TNF antagonist failures, during the maintenance study. PSDW was defined as an increase in pMCS of ≥3 to at least 5 points from the week 10 value at two consecutive visits, or an increase to 9 points at two consecutive visits if the week 10 value was >6. Bio, biologic; CI, confidence interval; HR, hazard ratio; MoA, mechanism of action; pMCS, partial Mayo Clinic Score; PBO, placebo; PSDW, protocol-specified disease worsening; TNF, tumour necrosis factor.

Time to PSDW was longer for patients who continued treatment with filgotinib 200 mg from induction through to maintenance, compared with patients who were re-randomized to placebo at maintenance baseline [Figure 3]. Filgotinib 200 mg-treated patients had a reduced risk of PSDW (HR [95% CI]) compared with patients who received placebo in both the biologic-naïve (0.22 [0.11–0.44]) and biologic-failed (0.22 [0.12–0.40]) groups [Figure 3A]. Among biologic-failed patients, those who received filgotinib 200 mg had a reduced risk of PSDW compared with patients who received placebo, independent of the number of previous biologic failures or biologic MoA failures: one biologic failure (0.18 [0.06–0.49]); more than one biologic failure (0.25 [0.11–0.55]); one biologic MoA failure (0.27 [0.11–0.64]); two MoA failures (0.17 [0.07–0.42]) [Figure 3B and C]. Specifically with respect to patients in whom TNF antagonists had failed, filgotinib 200 mg-treated patients with one previous failure had a reduced risk of PSDW compared with patients who received placebo, whereas those with more than one failure did not (0.13 [0.04–0.44] and 0.91 [0.13–6.31], respectively) [Figure 3D]. The probability of PSDW during maintenance generally increased with an increase in the number of failed biologics, biologic MoA classes and TNF antagonists, both for patients remaining on filgotinib 200 mg during maintenance and for those re-randomized to placebo [Figure 3].

Biologic-failed patients treated with filgotinib 100 mg had a reduced risk of PSDW compared with those who received placebo (0.40 [0.21–0.74]) [Supplementary Figure 3A]. Filgotinib 100 mg-treated patients in the biologic-failed subgroups of one biologic failure, two biologic MoA failures and one TNF antagonist failure also had a reduced risk of PSDW compared with patients who received placebo (0.16 [0.06–0.44], 0.21 [0.08–0.61] and 0.22 [0.07–0.71], respectively) [Supplementary Figure 3B–D]. A similar trend to that observed in patients remaining on filgotinib 200 mg, in terms of the likelihood of PSDW increasing with an increasing number of failed biologic MoAs, was observed in patients who received filgotinib 100 mg during induction, both in patients who continued to receive filgotinib treatment in the maintenance study and in those who were re-randomized to placebo [Supplementary Figure 3C].

### 3.5. Change in pMCS from induction baseline through to week 58

**Figure 4.**
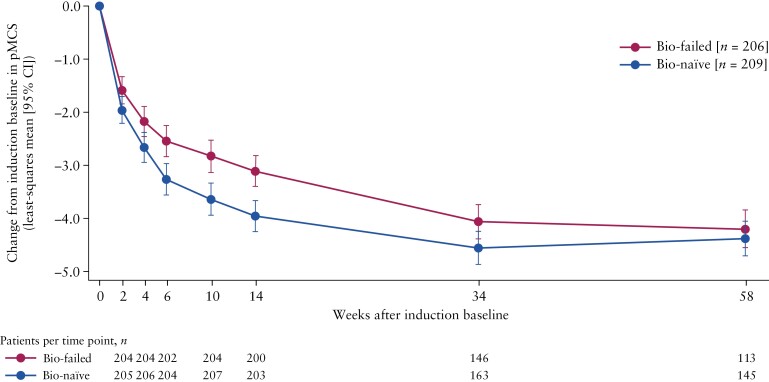
Least-squares mean [95% CI] change from induction baseline in pMCS for biologic-naïve and biologic-failed patients from the patient population that combined patients treated with filgotinib 200 mg throughout SELECTION and filgotinib 200 mg non-responders who entered SELECTIONLTE. Bio, biologic; CI, confidence interval; pMCS, partial Mayo Clinic Score.

In the patient population that combined patients treated with filgotinib 200 mg throughout SELECTION and filgotinib 200 mg non-responders, both the biologic-naïve and the biologic-failed groups achieved a reduction in mean pMCS from baseline that was maintained for 58 weeks [Figure 4]. As early as week 6, as well as at the end of induction (week 10), the biologic-failed patient group achieved a numerically larger reduction in mean pMCS than the biologic-naïve group. During maintenance, at both week 34 and week 58, the observed reduction was similar between the two subgroups.

## 4. Discussion

These *post hoc* analyses aimed to explore the effect of prior biologic therapy on the efficacy of filgotinib 200 mg in patients with moderately to severely active UC. Overall, the data indicate that filgotinib 200 mg was effective relative to placebo for the induction and maintenance of clinical remission in both biologic-naïve and biologic-failed patients.

At the end of induction, week 10, clinical remission and MCS response data indicated a treatment effect of filgotinib 200 mg in both biologic-naïve and biologic-failed patients. However, higher clinical remission rates were observed for filgotinib 200 mg in biologic-failed patients who had previously been treated with only one biologic or biologic MoA class than in those treated with multiple previous biologics or biologic MoA classes. Patients for whom multiple biologics had failed generally had slightly higher disease activity at baseline than those with failure of only one biologic or biologic MoA class, as evidenced by a higher baseline MES. It is known that a higher burden of disease in UC is associated with a higher rate of therapeutic failure.^15^ The selection of covariates for the logistic regression model we used to calculate ORs was based on an analysis of predictors of response to filgotinib.^16^ MES, however, was not found to be significant in the multivariate analysis for predictors of MCS response and/or clinical remission at week 10 and accordingly was not included in the present logistic regression model. Biologic failure status was identified as a predictor of response to filgotinib,^16^ and as a result, this factor may influence the lower response rates observed in patients with failure of multiple biologics, independent of baseline inflammatory burden. In addition, lower placebo response rates were observed in the biologic-failed subgroups as a function of increasing numbers of previous biologics and biologic MoA classes failed, indicating that these patients are increasingly treatment-refractory. Although there were differences in the treatment effect between groups at week 10, following treatment with filgotinib 200 mg, the rapidity of response, as assessed by RB subscore, was similar in the biologic-naïve and biologic-failed patient groups. This observation is consistent with previously published data on rapidity of response that showed that patients receiving filgotinib 200 mg had a rapid improvement in signs and symptoms of UC.^17^ In accordance with the observations made during induction, filgotinib 200 mg demonstrated beneficial effects as measured by clinical remission and MCS response at week 58 in both biologic-naïve and biologic-failed patient groups.

The absolute risk of PSDW during maintenance was lowest for biologic-naïve patients, higher in patients with failure of one biologic, and highest in those with failure of multiple biologics or biologic MoA classes. It is well established that patients with prior primary non-response or secondary loss of response are less likely to respond to a second biologic.^18^ Accordingly, it is clinically relevant that filgotinib 200 mg-treated patients were less likely to experience PSDW compared with placebo, with no evidence for the treatment effect being affected by the number of previously failed biologics or biologic MoAs. This suggests a sustained treatment benefit of filgotinib 200 mg among patients who responded during induction, regardless of previous treatment. Importantly, these data cannot be extrapolated to other treatment groups given the limitations that are inherent to the SELECTION randomized withdrawal study design. These limitations include that only induction responders continued maintenance treatment and that patients who were re-randomized to placebo after induction with filgotinib may have benefited from the residual effects of filgotinib.^13^

Data also suggest that filgotinib 100 mg provided some benefit as a maintenance dose in the biologic-naïve group, as these patients had the numerically lowest probability of PSDW at the end of the maintenance study. Additionally, biologic-failed patients treated with filgotinib 100 mg in the subgroups of one biologic failure, two biologic MoA failures and one TNF antagonist failure experienced a reduced risk of PSDW relative to placebo. The modest but detectable benefits of 100 mg in both biologic-naïve and biologic-experienced patients may suggest that induction with filgotinib 200 mg, followed by maintenance with filgotinib 100 mg could be effective in patients who require dose reduction. Nonetheless, data regarding the efficacy of filgotinib 100 mg should be interpreted cautiously given that dose de-escalation was not formally evaluated in the SELECTION trial. For this reason, the ongoing CAPYBARA trial [ClinicalTrials.gov ID: NCT05479058] is evaluating the comparative efficacy and safety of dose de-escalation to filgotinib 100 mg relative to maintaining the 200 mg dose, for patients who achieve remission with filgotinib 200 mg in SELECTIONLTE.^19^

Finally, in a patient population that included induction non-responders who entered SELECTIONLTE, patients receiving filgotinib 200 mg in the biologic-naïve and biologic-failed groups had a similar reduction in pMCS from induction baseline through to week 58. At week 10, the biologic-naïve group had a reduction in pMCS that was statistically significantly larger than that seen in the biologic-failed group. However, during maintenance, the observed reductions in pMCS converged between the two groups; this effect may have been influenced by patients discontinuing the study, particularly from the biologic-failed group. Although this analysis also included patients who responded to filgotinib 200 mg during induction, this finding may suggest that filgotinib provides some benefit in patients who did not initially respond during induction, both without prior biologic usage and with prior biologic failure.

It is also relevant to consider the potential effects of prior biologic usage on patient safety. Integrated safety data from SELECTION and SELECTIONLTE show that filgotinib 200 mg was well tolerated and had an acceptable safety profile in patients with moderately to severely active UC, in both biologic-naïve patients and in patients in whom biological therapy failed.^20^ Patients receiving filgotinib 200 mg who had a history of biologic use had an increased incidence of treatment-emergent infections compared with those receiving filgotinib 200 mg as first-line therapy,^20^ which may be related to the present findings that suggest that the biologic-failed group is particularly difficult to treat.

The strengths of these *post hoc* analyses include the logistic regression model used to calculate ORs for clinical remission and MCS response, the Cox proportional hazards model used to calculate HRs for time to PSDW, and the MMRM used to calculate the least-squares mean change in pMCS from baseline, all of which accounted for relevant covariates identified as predictors of response to filgotinib.

These analyses do, however, have some limitations. For example, there is an inherent bias in the population investigated for PSDW, because only patients with a response at week 10 were re-randomized into the maintenance study. Furthermore, these are *post hoc* rather than pre-specified analyses. These *post hoc* analyses resulted in patient numbers in the biologic-failed subgroups that were sometimes small, particularly for the maintenance study, and any reported statistical significance herein was nominal. Additionally, endpoints used in the trial may not be the same as in clinical practice. For instance, the PSDW endpoint assessed here may not have been based on the same criteria as those used to terminate treatment in real-world clinical practice, given that patients are considered on an individual basis in real-world settings. Moreover, although these *post hoc* analyses provide some clinical evidence for the positioning of filgotinib therapy in UC, not all biologics and small molecules were included as part of the study design. Likewise, the population of patients who had exclusively failed vedolizumab was too small to allow statistically meaningful analyses of clinical remission, MCS response and PSDW. Finally, given that the SELECTION study population was predominantly White and not Hispanic or Latino, these results may not be fully generalizable to other populations. Building on the findings presented here, future studies should further examine the predictors of response to filgotinib in biologic-naïve and biologic-failed patients.

Overall, these data support the efficacy of filgotinib 200 mg as induction and maintenance therapy for UC regardless of treatment history, with the greatest sustained benefits observed when filgotinib is used as a first-line therapy before biologics or as a second-line therapy after failure of only one biologic or biologic MoA class.

## Supplementary Material

jjad039_suppl_Supplementary_Material

## Data Availability

Anonymized individual patient data will be shared upon request for research purposes dependent upon the nature of the request, the merit of the proposed research, the availability of the data and its intended use. The full data sharing policy for Gilead Sciences, Inc., can be found at https://www.gileadclinicaltrials.com/transparency-policy/.

## References

[1] Ungaro R , Mehandru S, Allen PB, Peyrin-Biroulet L, Colombel JF. Ulcerative colitis. Lancet 2017;389:1756–70. 27914657 10.1016/S0140-6736(16)32126-2 PMC6487890

[2] Turner D , Ricciuto A, Lewis A, et al; International Organization for the Study of IBD. STRIDE-II: an update on the selecting therapeutic targets in inflammatory bowel disease (STRIDE) initiative of the international organization for the study of IBD (IOIBD): determining therapeutic goals for treat-to-target strategies in IBD. Gastroenterology 2021;160:1570–83. 33359090 10.1053/j.gastro.2020.12.031

[3] Kim JW , Kim SY. The era of janus kinase inhibitors for inflammatory bowel disease treatment. Int J Mol Sci 2021;22:11322. 34768752 10.3390/ijms222111322 PMC8582842

[4] Baumgart DC , Le Berre C. Newer biologic and small-molecule therapies for inflammatory bowel disease. N Engl J Med 2021;385:1302–15. 34587387 10.1056/NEJMra1907607

[5] European Medicines Agency. *Jyseleca (filgotinib) EPAR - product information*. 2022. https://www.ema.europa.eu/en/documents/product-information/jyseleca-epar-product-information_en.pdf Accessed July 1, 2022.

[6] Sanachai K , Mahalapbutr P, Choowongkomon K, Poo-Arporn RP, Wolschann P, Rungrotmongkol T. Insights into the binding recognition and susceptibility of tofacitinib toward janus kinases. ACS Omega 2020;5:369–77. 31956784 10.1021/acsomega.9b02800 PMC6964278

[7] Biggioggero M , Becciolini A, Crotti C, Agape E, Favalli EG. Upadacitinib and filgotinib: the role of JAK1 selective inhibition in the treatment of rheumatoid arthritis. Drugs Context 2019;8:212595. 31692920 10.7573/dic.212595 PMC6821397

[8] Traves PG , Murray B, Campigotto F, Galien R, Meng A, Di Paolo JA. JAK selectivity and the implications for clinical inhibition of pharmacodynamic cytokine signalling by filgotinib, upadacitinib, tofacitinib and baricitinib. Ann Rheum Dis 2021;80:865–75. 33741556 10.1136/annrheumdis-2020-219012 PMC8237188

[9] Singh S , Fumery M, Sandborn WJ, Murad MH. Systematic review with network meta-analysis: first- and second-line pharmacotherapy for moderate–severe ulcerative colitis. Aliment Pharmacol Ther 2018;47:162–75. 29205406 10.1111/apt.14422

[10] Peyrin-Biroulet L , Sandborn WJ, Panaccione R, et al. Tumour necrosis factor inhibitors in inflammatory bowel disease: the story continues. Therap Adv Gastroenterol 2021;14:17562848211059954. 10.1177/17562848211059954 PMC8669878 34917173

[11] Rubin DT , Ananthakrishnan AN, Siegel CA, Sauer BG, Long MD. ACG clinical guideline: ulcerative colitis in adults. Am J Gastroenterol 2019;114:384–413. 30840605 10.14309/ajg.0000000000000152

[12] Sands BE , Cheifetz AS, Nduaka CI, et al. The impact of raising the bar for clinical trials in ulcerative colitis. J Crohns Colitis 2019;13:1217–26. 30879034 10.1093/ecco-jcc/jjz038 PMC6821359

[13] Feagan BG , Danese S, Loftus EV Jr, et al. Filgotinib as induction and maintenance therapy for ulcerative colitis (SELECTION): a phase 2b/3 double-blind, randomised, placebo-controlled trial. Lancet 2021;397:2372–84. 34090625 10.1016/S0140-6736(21)00666-8

[14] Galapagos NV. *Filgotinib in long-term extension study of adults with ulcerative colitis (SELECTIONLTE)* . 2016. https://clinicaltrials.gov/ct2/show/NCT02914535 Accessed January 21, 2022.

[15] Silva JC , Fernandes C, Rodrigues J, et al. Endoscopic and histologic activity assessment considering disease extent and prediction of treatment failure in ulcerative colitis. Scand J Gastroenterol 2020;55:1157–62. 32772587 10.1080/00365521.2020.1803397

[16] Feagan B , Peyrin-Biroulet L, Louis E, et al. Predictors of response to filgotinib in ulcerative colitis: *post hoc* analysis from the SELECTION study [abstract]. United European Gastronterol J 2022;10:S462–3.

[17] Danese S , Ferrante M, Feagan BG, et al. Rapid and sustained symptom relief in patients with ulcerative colitis treated with filgotinib: data from the phase 2b/3 SELECTION trial. Am J Gastroenterol 2023;118:138–47. 36113491 10.14309/ajg.0000000000001979 PMC9810009

[18] Singh S , George J, Boland BS, Vande Casteele N, Sandborn WJ. Primary non-response to tumor necrosis factor antagonists is associated with inferior response to second-line biologics in patients with inflammatory bowel diseases: a systematic review and meta-analysis. J Crohns Colitis 2018;12:635–43. 29370397 10.1093/ecco-jcc/jjy004 PMC7189966

[19] Galapagos NV. *A study evaluating the effect of filgotinib dose de-escalation in participants with ulcerative colitis (UC) in remission (CAPYBARA)* . 2022. https://clinicaltrials.gov/ct2/show/NCT05479058 Accessed August 4, 2022.

[20] Schreiber S , Wantanabe M, Yun C, et al. Safety analysis of filgotinib for ulcerative colitis: results from the phase 2b/3 SELECTION study and phase 3 SELECTION LTE long-term extension study [abstract]. United European Gastroenterol. J 2021;9:S76.

