# Supplementary material for: Efficacy of Filgotinib in Patients with Ulcerative Colitis by Line of Therapy in the Phase 2b/3 SELECTION Trial
Source: J Crohns Colitis. 2023 Mar 16;17(8):1207–16. doi: 10.1093/ecco-jcc/jjad039 (PMC10441561; doi:10.1093/ecco-jcc/jjad039)
Supplement: jjad039_suppl_Supplementary_Material [file jjad039_suppl_supplementary_material.docx]

Supplementary Material

**Supplementary Figure 1.** SELECTION trial design


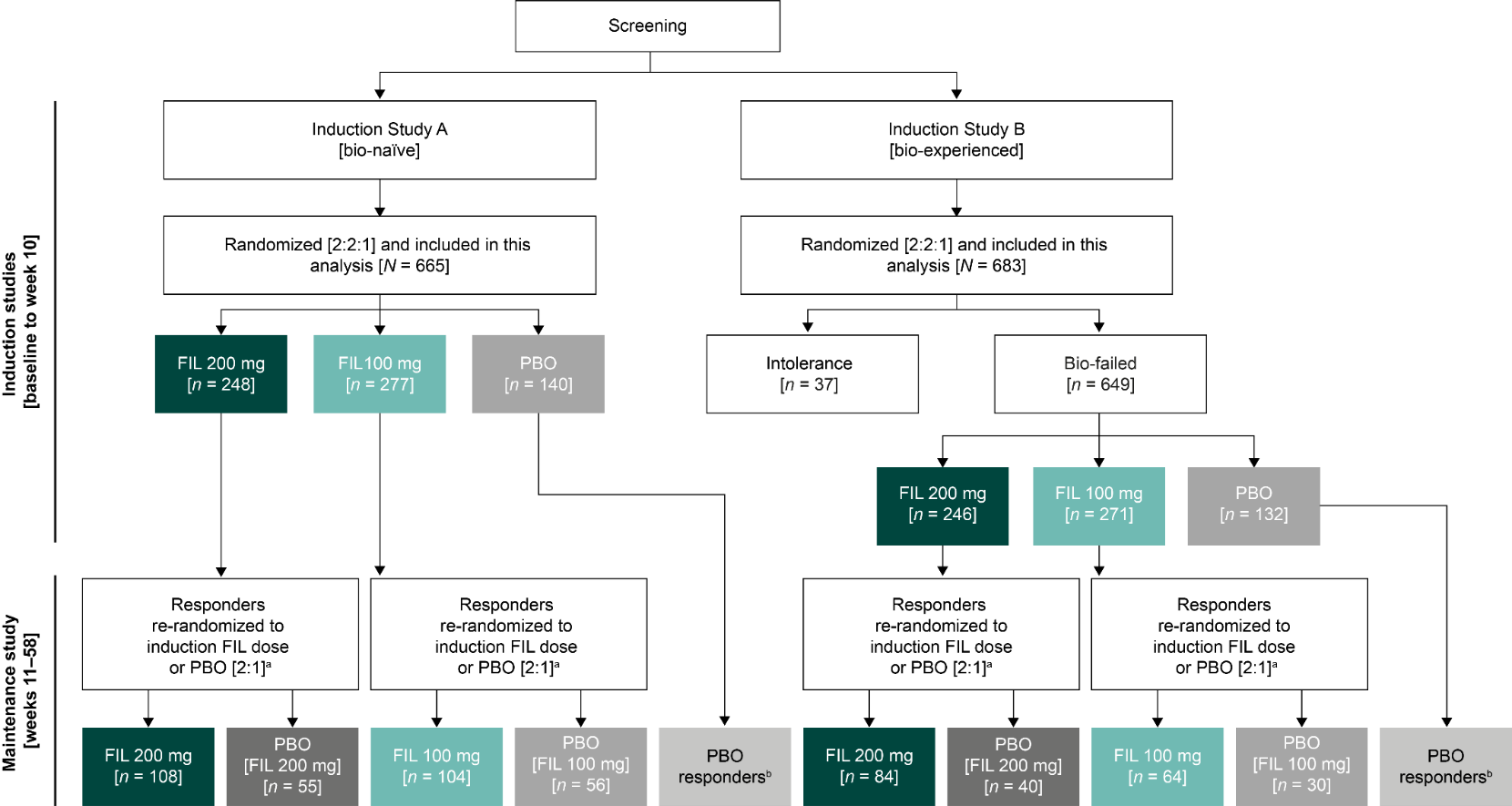


^a^Responders were defined as patients who received filgotinib and achieved either clinical remission or a MCS response at week 10. Clinical remission was defined as a MES of 0 or 1, RB subscore of 0, and a ≥1 point decrease in SF subscore from induction baseline to achieve a subscore of 0 or 1. A MCS response was defined as a reduction of ≥3 points in MCS that resulted in a MCS score that was ≥30% smaller compared with induction baseline, with an accompanying decrease in RB subscore of ≥1 point, or an absolute RB subscore of 0 or 1

^b^PBO responders continued receiving PBO in maintenance and were not considered in this post hoc analysis of the SELECTION trial

bio, biologic; FIL, filgotinib; MCS, Mayo clinic score; MES, Mayo endoscopic score; PBO, placebo; RB, rectal bleeding; SF, stool frequency

**Supplementary Figure 2.** Proportions of biologic-naïve and biologic-failed patients in [A] clinical remission and [B] with a MCS response at week 58, and proportions of patients by number of failed biologics, MoA classes and TNF antagonists in [C] clinical remission and [D] with a MCS response at week 58.


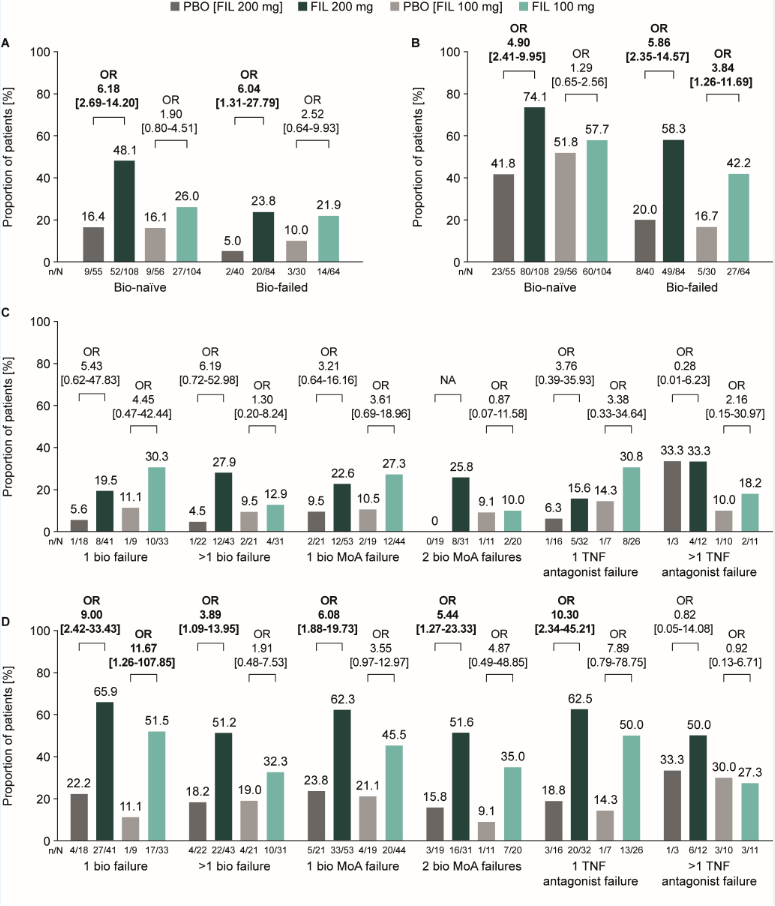


ORs [95% CI] are given in terms of FIL 100mg or FIL 200mg versus placebo in each of the groups and subgroups. ORs [95% CI] given in **bold** indicate statistical significance.

The OR [95% CI] of FIL 200mg versus placebo in the 2 MoA failures subgroup could not be calculated as no patients re-randomized to placebo from FIL 200mg had clinical remission.

Bio, biologic; FIL, filgotinib; MCS, Mayo Clinic Score; MoA, mechanism of action; NA, not available; OR, odds ratio; PBO, placebo; TNF, tumour necrosis factor.

**Supplementary Figure 3.** Time to PSDW for patients treated with filgotinib 100 mg during induction in [A] biologic-naïve and biologic-failed patients, and among biologic-failed patients categorized by number of [B] biologic failures, [C] MoA class failures, and [D] TNF antagonist failures, during the maintenance study.


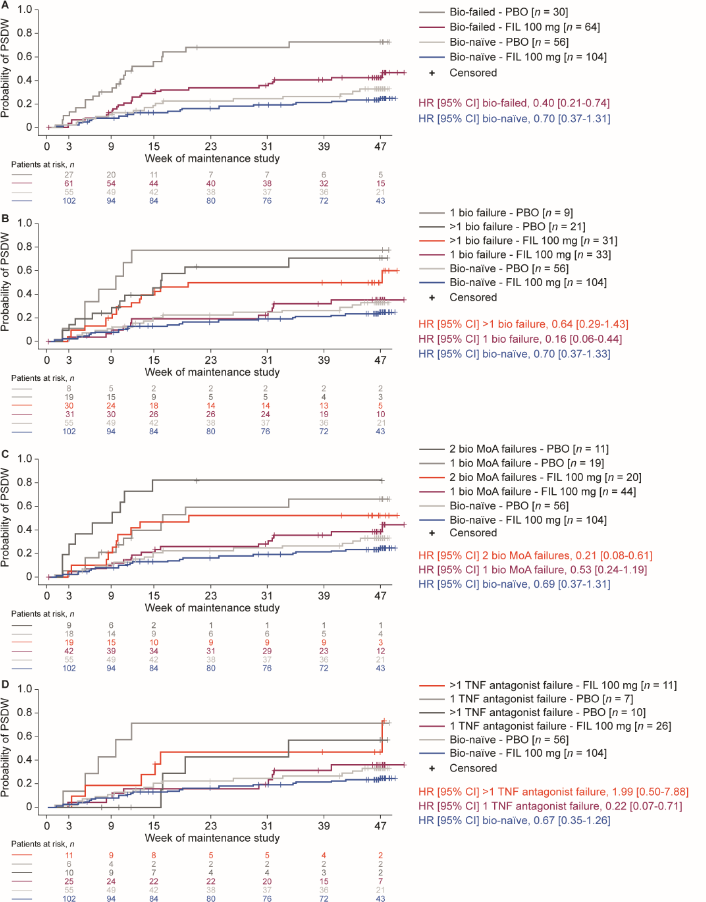


PSDW was defined as an increase in pMCS of ≥3 to at least 5 points from the week 10 value at two consecutive visits, or an increase to 9 points at 2 consecutive visits if the week 10 value was >6.

Bio, biologic; CI, confidence interval; HR, hazard ratio; MoA, mechanism of action; pMCS, partial Mayo Clinic Score; PBO, placebo; PSDW, protocol-specified disease worsening; TNF, tumour necrosis factor.

**Supplementary Table 1.** Patient induction baseline and disease characteristics for the biologic-failed subgroups.

|  | **Failure of 1 biologic^a^** | | | **Failure of >1 biologic** | | |
| --- | --- | --- | --- | --- | --- | --- |
|  | **Filgotinib  200 mg**  **[*n* = 98]** | **Filgotinib  100 mg**  **[*n* = 112]** | **Placebo**  **[*n* = 51]** | **Filgotinib  200 mg**  **[*n* = 148]** | **Filgotinib  100 mg**  **[*n* = 159]** | **Placebo**  **[*n* = 81]** |
| Age, years, mean ± SD | 45.4 **±** 15.3 | 42.6 **±** 14.3 | 42.5 **±** 15.0 | 42.1 **±** 13.2 | 43.0 **±** 14.3 | 45.2 **±** 15.2 |
| Sex, female, n [%] | 50 [51.0] | 39 [34.8] | 21 [41.2] | 52 [35.1] | 52 [32.7] | 31 [38.3] |
| Race, n [%]  American Indian or Alaska native  Asian  Black or African American  White  Other  Not permitted^b^ | 0  29 [29.6]  2 [2.0]  65 [66.3]  0  2 [2.0] | 0  23 [20.5]  4 [3.6]  82 [73.2]  0  3 [2.7] | 0  13 [25.5]  2 [3.9]  33 [64.7]  1 [2.0]  2 [3.9] | 0  17 [11.5]  2 [1.4]  113 [76.4]  0  16 [10.8] | 0  22 [13.8]  2 [1.3]  122 [76.7]  0  13 [8.2] | 0  13 [16.0]  1 [1.2]  57 [70.4]  0  10 [12.3] |
| Ethnicity, n [%]  Not Hispanic or Latino  Hispanic or Latino  Not permitted^b^ | 94 [95.9]  3 [3.1]  1 [1.0] | 109 [97.3]  3 [2.7]  0 | 48 [94.1]  3 [5.9]  0 | 140 [94.6]  4 [2.7]  4 [2.7] | 150 [94.3]  5 [3.1]  4 [2.5] | 76 [93.8]  1 [1.2]  4 [4.9] |
| Smoking status, n [%]  Former  Current  Never | 31 [31.6]  2 [2.0]  65 [66.3] | 33 [29.5]  8 [7.1]  71 [63.4] | 12 [23.5]  3 [5.9]  36 [70.6] | 38 [25.7]  5 [3.4]  105 [70.9] | 56 [35.2]  12 [7.5]  91 [57.2] | 29 [35.8]  2 [2.5]  50 [61.7] |
| Duration of UC from diagnosis, years, mean ± SD | 9.4 **±** 7.8 | 8.6 **±** 6.8 | 11.7 **±** 9.7 | 10.0 **±** 7.3 | 10.4 **±** 7.3 | 9.1 **±** 7.2 |
| MCS, mean ± SD | 9.0 **±** 1.5 | 9.1 **±** 1.3 | 9.1 **±** 1.6 | 9.5 **±** 1.3 | 9.4 **±** 1.2 | 9.4 **±** 1.3 |
| Mayo endoscopic subscore of 3, n [%] | 70 [71.4] | 83 [74.1] | 38 [74.5] | 123 [83.1] | 130 [81.8] | 66 [81.5] |
| Fecal calprotectin, μg/g, mean ± SD | 2746 ± 4095 | 2273 ± 3024 | 2828 ± 3846 | 2867 ± 3959 | 2459 ± 3870 | 2338 ± 3565 |
| Concomitant use of corticosteroids on day 1, n [%] | 43 [43.9] | 44 [39.3] | 19 [37.3] | 74 [50.0] | 79 [49.7] | 41 [50.6] |
| Prior use of adalimumab, n [%] | 42 [42.9] | 41 [36.6] | 15 [29.4] | 88 [59.5] | 101 [63.5] | 57 [70.4] |
| Prior use of golimumab, n [%] | 10 [10.2] | 11 [9.8] | 3 [5.9] | 46 [31.1] | 43 [27.0] | 24 [29.6] |
| Prior use of infliximab, n [%] | 51 [52.0] | 61 [54.5] | 30 [58.8] | 133 [89.9] | 141 [88.7] | 66 [81.5] |
| Prior use of vedolizumab, n [%] | 35 [35.7] | 26 [23.2] | 17 [33.3] | 127 [85.8] | 116 [73.0] | 66 [81.5] |
| Prior failure of adalimumab, n [%] | 32 [32.7] | 37 [33.0] | 15 [29.4] | 87 [58.8] | 96 [60.4] | 54 [66.7] |
| Prior failure of golimumab, n [%] | 5 [5.1] | 8 [7.1] | 3 [5.9] | 44 [29.7] | 42 [26.4] | 23 [28.4] |
| Prior failure of infliximab, n [%] | 33 [33.7] | 48 [42.9] | 21 [41.2] | 120 [81.1] | 128 [80.5] | 59 [72.8] |
| Prior failure of vedolizumab, n [%] | 24 [24.5] | 14 [12.5] | 10 [19.6] | 120 [81.1] | 114 [71.7] | 64 [79.0] |
| Prior use of both TNF antagonists^c^  and vedolizumab, n [%] | 19 [19.4] | 11 [9.8] | 9 [17.6] | 127 [85.8] | 116 [73.0] | 66 [81.5] |
| Prior failure of both TNF antagonists^c^ and vedolizumab, n [%] | 0 | 0 | 0 | 120 [81.1] | 114 [71.7] | 64 [79.0] |

|  | **Failure of 1 MoA** | | | **Failure of 2 MoAs** | | | **Failure of 1 TNF antagonist** | | | **Failure of >1 TNF antagonist** | | |
| --- | --- | --- | --- | --- | --- | --- | --- | --- | --- | --- | --- | --- |
|  | **Filgotinib  200 mg**  **[*n* = 126]** | **Filgotinib  100 mg**  **[*n* = 157]** | **Placebo**  **[*n* = 68]** | **Filgotinib  200 mg**  **[*n* = 120]** | **Filgotinib  100 mg**  **[*n* = 114]** | **Placebo**  **[*n* = 64]** | **Filgotinib  200 mg**  **[*n* = 70]** | **Filgotinib  100 mg**  **[*n* = 93]** | **Placebo**  **[*n* = 39]** | **Filgotinib  200 mg**  **[*n* = 28]** | **Filgotinib  100 mg**  **[*n* = 45]** | **Placebo**  **[*n* = 17]** |
| Age, years, mean ± SD | 44.4 **±** 15.0 | 42.4 **±** 14.2 | 44.5 **±** 15.1 | 42.4 **±** 13.2 | 43.4 **±** 14.3 | 43.8 **±** 15.2 | 45.5 ± 15.0 | 41.0 ± 14.1 | 41.1 ± 15.9 | 40.9 ± 13.4 | 42.0 ± 14.1 | 50.5 ± 14.3 |
| Sex, female, n [%] | 61 [48.4] | 52 [33.1] | 24 [35.3] | 41 [34.2] | 39 [34.2] | 28 [43.8] | 38 [54.3] | 31 [33.3] | 17 [43.6] | 11 [39.3] | 13 [28.9] | 3 [17.6] |
| Race, n [%]  American Indian or Alaska native  Asian  Black or African American  White  Other  Not permitted^b^ | 0  39 [31.0]  3 [2.4]  80 [63.5]  0  4 [3.2] | 0  40 [25.5]  5 [3.2]  108 [68.8]  0  4 [2.5] | 0  20 [29.4]  3 [4.4]  41 [60.3]  1 [1.5]  3 [4.4] | 0  7 [5.8]  1 [0.8]  98 [81.7]  0  14 [11.7] | 0  5 [4.4]  1 [0.9]  96 [84.2]  0  12 [10.5] | 0  6 [9.4]  0  49 [76.6]  0  9 [14.1] | 0  27 [38.6]  1 [1.4]  42 [60.0]  0  0 | 0  21 [22.6]  4 [4.3]  65 [69.9]  0  3 [3.2] | 0  12 [30.8]  2 [5.1]  22 [56.4]  1 [2.6]  2 [5.1] | 0  10 [35.7]  1 [3.6]  15 [53.6]  0  2 [7.1] | 0  17 [37.8]  1 [2.2]  26 [57.8]  0  1 [2.2] | 0  7 [41.2]  1 [5.9]  8 [47.1]  0  1 [5.9] |
| Ethnicity, n [%]  Not Hispanic or Latino  Hispanic or Latino  Not permitted^b^ | 120 [95.2]  4 [3.2]  2 [1.6] | 151 [96.2]  6 [3.8]  0 | 63 [92.6]  4 [5.9]  1 [1.5] | 114 [95.0]  3 [2.5]  3 [2.5] | 108 [94.7]  2 [1.8]  4 [3.5] | 61 [95.3]  0  3 [4.7] | 67 [95.7]  3 [4.3]  0 | 91 [97.8]  2 [2.2]  0 | 36 [92.3]  3 [7.7]  0 | 26 [92.9]  1 [3.6]  1 [3.6] | 42 [93.3]  3 [6.7]  0 | 15 [88.2]  1 [5.9]  1 [5.9] |
| Smoking status, n [%]  Former  Current  Never | 42 [33.3]  2 [1.6]  82 [65.1] | 53 [33.8]  10 [6.4]  94 [59.9] | 19 [27.9]  3 [4.4]  46 [67.6] | 27 [22.5]  5 [4.2]  88 [73.3] | 36 [31.6]  10 [8.8]  68 [59.6] | 22 [34.4]  2 [3.1]  40 [62.5] | 23 [32.9]  2 [2.9]  45 [64.3] | 28 [30.1]  7 [7.5]  58 [62.4] | 9 [23.1]  2 [5.1]  28 [71.8] | 11 [39.3]  0  17 [60.7] | 20 [44.4]  2 [4.4]  23 [51.1] | 7 [41.2]  0  10 [58.8] |
| Duration of UC from diagnosis, years, mean ± SD | 9.6 **±** 7.3 | 9.3 **±** 6.7 | 11.5 **±** 9.4 | 9.9 **±** 7.7 | 10.1 **±** 7.6 | 8.7 **±** 6.7 | 8.8 ± 6.6 | 8.6 ± 6.8 | 11.2 ± 10.4 | 10.2 ± 5.4 | 11.0 ± 6.4 | 10.9 ± 8.9 |
| MCS, mean ± SD | 9.0 **±** 1.4 | 9.2 **±** 1.3 | 9.2 **±** 1.6 | 9.5 **±** 1.3 | 9.5 **±** 1.2 | 9.4 **±** 1.3 | 8.8 ± 1.5 | 9.1 ± 1.4 | 9.2 ± 1.6 | 9.3 ± 1.3 | 9.3 ± 1.3 | 9.5 ± 1.6 |
| Mayo endoscopic subscore of 3, n [%] | 95 [75.4] | 119 [75.8] | 53 [77.9] | 98 [81.7] | 94 [82.5] | 51 [79.7] | 47 [67.1] | 65 [69.9] | 29 [74.4] | 25 [89.3] | 36 [80.0] | 15 [88.2] |
| Fecal calprotectin, μg/g, mean ± SD | 2804 ± 4017 | 2148 ± 2834 | 2666 ± 3465 | 2833 ± 4011 | 2696 ± 4302 | 2382 ± 3913 | 2779 ± 4362 | 2395 ± 3235 | 3093 ± 4286 | 3013 ± 3794 | 1821 ± 2263 | 2180 ± 1932 |
| Concomitant use of corticosteroids on day 1, n [%] | 54 [42.9] | 68 [43.3] | 26 [38.2] | 63 [52.5] | 55 [48.2] | 34 [53.1] | 33 [47.1] | 37 [39.8] | 16 [41.0] | 11 [39.3] | 24 [53.3] | 7 [41.2] |
| Prior use of adalimumab, n [%] | 63 [50.0] | 77 [49.0] | 31 [45.6] | 67 [55.8] | 65 [57.0] | 41 [64.1] | 37 [52.9] | 40 [43.0] | 15 [38.5] | 21 [75.0] | 36 [80.0] | 16 [94.1] |
| Prior use of golimumab, n [%] | 24 [19.0] | 28 [17.8] | 7 [10.3] | 32 [26.7] | 26 [22.8] | 20 [31.3] | 8 [11.4] | 9 [9.7] | 3 [7.7] | 14 [50.0] | 17 [37.8] | 4 [23.5] |
| Prior use of infliximab, n [%] | 78 [61.9] | 101 [64.3] | 46 [67.6] | 106 [88.3] | 101 [88.6] | 50 [78.1] | 42 [60.0] | 58 [62.4] | 26 [66.7] | 27 [96.4] | 40 [88.9] | 16 [94.1] |
| Prior use of vedolizumab, n [%] | 42 [33.3] | 28 [17.8] | 19 [27.9] | 120 [100] | 114 [100] | 64 [100] | 7 [10.0] | 7 [7.5] | 5 [12.8] | 7 [25.0] | 2 [4.4] | 2 [11.8] |
| Prior failure of adalimumab, n [%] | 53 [42.1] | 72 [45.9] | 30 [44.1] | 66 [55.0] | 61 [53.5] | 39 [60.9] | 32 [45.7] | 37 [39.8] | 15 [38.5] | 21 [75.0] | 35 [77.8] | 15 [88.2] |
| Prior failure of golimumab, n [%] | 19 [15.1] | 25 [15.9] | 7 [10.3] | 30 [25.0] | 25 [21.9] | 19 [29.7] | 5 [7.1] | 8 [8.6] | 3 [7.7] | 14 [50.0] | 17 [37.8] | 4 [23.5] |
| Prior failure of infliximab, n [%] | 58 [46.0] | 88 [56.1] | 37 [54.4] | 95 [79.2] | 88 [77.2] | 43 [67.2] | 33 [47.1] | 48 [51.6] | 21 [53.8] | 25 [89.3] | 40 [88.9] | 16 [94.1] |
| Prior failure of vedolizumab, n [%] | 24 [19.0] | 14 [8.9] | 10 [14.7] | 120 [100] | 114 [100] | 64 [100] | 0 | 0 | 0 | 0 | 0 | 0 |
| Prior use of both TNF antagonists^c^  and vedolizumab, n [%] | 26 [20.6] | 13 [8.3] | 11 [16.2] | 120 [100] | 114 [100] | 64 [100] | 7 [10.0] | 7 [7.5] | 5 [12.8] | 7 [25.0] | 2 [4.4] | 2 [11.8] |
| Prior failure of both TNF antagonists^c^ and vedolizumab, n [%] | 0 | 0 | 0 | 120 [100] | 114 [100] | 64 [100] | 70 [100] | 93 [100] | 39 [100] | 28 [100] | 45 [100] | 17 [100] |

^a^In the 1 biologic failure group, a small number of patients in each treatment arm (filgotinib 200 mg: n = 4; filgotinib 100 mg: n = 5; placebo: n = 2) had ‘vedolizumab/adalimumab (blinded study medication)’ as their recorded single previous failure of a biologic therapy. Accordingly, data on the prior failure of the individual TNFs and vedolizumab was not available for these patients.

^b^Local regulators did not allow collection of race and/or ethnicity information.

^c^TNF antagonists included adalimumab, golimumab and infliximab.

MCS, Mayo Clinic Score; MoA, mechanism of action; SD, standard deviation; TNF, tumour necrosis factor; UC, ulcerative colitis.

Plain language summary

**Efficacy of filgotinib in patients with ulcerative colitis by line of therapy in the phase 2b/3 SELECTION trial**

Iris Dotan, Brian G. Feagan, Virginia Taliadouros_,_ Alessandra Oortwijn, Christine Rudolph, Angela de Haas, Eva Santermans, Jeremy Hsieh, Laurent Peyrin-Biroulet, Toshifumi Hibi

Ulcerative colitis (UC) is an inflammatory bowel disease affecting the gut lining.
Multiple UC treatments are available, but patients may not respond to their first treatment. Previous treatments may also change how effective the next treatment is. The SELECTION trial compared 200 mg filgotinib with placebo in adults with moderately to severely active UC over 58 weeks. Filgotinib works by blocking Janus kinase 1, an intracellular protein involved in the inflammatory signalling processes in UC. This analysis from SELECTION looked at the probability of a patient having clinical remission 10 weeks after treatment. Clinical remission meant having no rectal bleeding, a reduced stool frequency to fewer than 3 stools per day more than normal, and mild or inactive UC disease based on gut lining scans. The probability that a patient had protocol-specific disease worsening (PSDW) from weeks 11 to 58 was also calculated. DW meant a patient’s pMCS (partial Mayo Clinic Score) increased by a specific amount compared with their pMCS at week 10. pMCS was calculated by assessing a patient’s rectal bleeding, stool frequency and overall UC disease. The analysis considered two groups: patients who never received biologic treatments (protein-based molecules) and patients for whom biologic treatments failed. The results showed that patients treated with filgotinib were more likely to have clinical remission at week 10 and less likely to have PSDW regardless of their biologic treatment history, compared with patients who received placebo. Patients who had more biologic treatments fail for them were more likely to have PSDW.

**Word count:** 250
